# Effects of Sphingosine 1-phosphate Modulators on Central Remyelination: A Systematic Review of Animal Models

**DOI:** 10.1007/s10571-026-01748-0

**Published:** 2026-06-10

**Authors:** Harley Vu, Nelson George, Junhua Xiao

**Affiliations:** Department of Biomedical, Health and Exercise Sciences, School of Health Sciences, Swinburne University of Technology, Hawthorn, VIC 3122 Australia

**Keywords:** S1P, Remyelination, Demyelination, Oligodendrocyte, Multiple sclerosis, In vivo

## Abstract

Promoting remyelination is a key therapeutic goal in demyelinating diseases such as multiple sclerosis (MS), yet effective strategies remain limited. Sphingosine-1-phosphate (S1P), a ubiquitous bioactive lipid, has emerged as a key therapeutic target in MS due to its dual roles in immune regulation and neuroprotection; however, the therapeutic efficacy of current S1P-based therapies in remyelination remains unclear. This systematic review evaluated in vivo studies up to July 2025, in accordance with PRISMA guidelines, to assess the efficacy of S1P modulators on remyelination in mammalian models of demyelination. A comprehensive search across three databases identified 24 eligible studies that investigated S1P receptor (S1PR) modulation in both acute and chronic models of demyelination, with or without immune-mediated components. Fingolimod was the most extensively studied compound (16 studies). Of the 18 studies assessing demyelination outcomes, S1P modulation consistently attenuated myelin loss and oligodendrocyte depletion. In contrast, remyelination outcomes were inconsistent: among 15 studies assessing repair, most reported no significant enhancement. While fingolimod showed limited evidence on remyelination, more promising effects were observed with selective S1PR1/5 modulators such as siponimod and ponesimod. Overall, current evidence supports a model in which S1P modulators act primarily through S1PR1-mediated immunomodulation and S1PR5-associated oligodendroglial protection, preserving oligodendrocyte lineage cells rather than driving terminal differentiation or de novo remyelination. Several compounds displayed bell-shaped dose–response patterns, highlighting the importance of dosing and treatment paradigms. Collectively, these findings indicate S1PR-based therapies primarily limit demyelination, with limited evidence of remyelination, emphasising the need for more efficacious S1P modulators to improve MS outcomes.

## Introduction

Myelin is a specialised, lipid-rich membrane that ensheathes many axons in the nervous system (Kister and Kister 2023; Stadelmann et al. 2019; Nave and Werner 2014), enabling rapid saltatory conduction and providing essential trophic support to neurons (Roggeri et al. 2020). Approximately 70–85% of myelin content is comprised of lipids, including cholesterol, phospholipids, and glycolipids, contributing to the structural stability and compaction of the myelin sheath (Poitelon et al. 2020). Demyelination, defined as the loss or damage of myelin, occurs in both genetic and acquired neurological diseases, disrupting axonal conduction and ultimately leading to progressive neurological disability (Coutinho Costa et al. 2023). Indeed, demyelination is a pathological hallmark of a plethora of central nervous system (CNS) disorders, particularly inflammatory demyelinating diseases such as multiple sclerosis (MS), neuromyelitis optica (NMO), and acute disseminated encephalomyelitis (ADEM) (Coutinho Costa et al. 2023; Pirko and Noseworthy 2007). In MS, persistent neuroinflammation eventually leads to irreversible axonal damage and neurodegeneration, emphasising that the prevention of demyelination alone is insufficient to maintain long-term neurological function (Dighriri et al. 2023; Haki et al. 2024). Although MS lesions possess an intrinsic capacity for remyelination early in the disease course, many lesions remain chronically demyelinated, leading to irreversible axonal degeneration that drives clinical deficits in later stages. Indeed, incomplete or failure of remyelination remains an obstacle to functional recovery in MS.

Remyelination is a complex, multicellular process influenced by axon-glia interactions, innate and systemic immune responses, and the extracellular milieu (Franklin and Simons 2022; Bourdette and Wooliscroft 2024). It begins with the clearance of myelin debris, followed by the activation of oligodendrocyte precursor cells (OPCs), which migrate to demyelinated regions and differentiate into myelinating oligodendrocytes (OLGs) (Leo and Kipp 2022; Kotter et al. 2006; Keirstead and Blakemore 1999). This lineage progression is accompanied by stage-specific molecular changes, with markers such as NG2 and PDGFRα identifying OPCs, O1, O4 and protein proteolipid (PLP) marking pre-oligodendrocytes, and CC1, CNPase, myelin-associated glycoprotein (MAG), or myelin basic protein (MBP) characterising mature myelinating cells, enabling the detailed assessment of remyelination dynamics in experimental models (Leo and Kipp 2022; Roggeri et al. 2020). Due to the complexity of these coordinated processes and the inflammatory microenvironment in myelin lesions, remyelination cannot be fully recapitulated in vitro. Therefore, in vivo mammalian models play a central role in remyelination research by enabling the assessment of efficacy within an intact CNS environment, where factors such as blood–brain barrier (BBB) permeability and cellular interactions can influence therapeutic outcomes (Lubetzki et al. 2020). Commonly used models such as cuprizone (CPZ), lysolecithin (LPC), and experimental autoimmune encephalomyelitis (EAE) mimic various pathophysiological aspects of MS and provide complementary information on toxin-induced demyelination, focal lesions, and autoimmune-driven neuropathology, respectively (Procaccini et al. 2015; Ransohoff 2012; Dedoni et al. 2023).

Over the past decade, significant progress has been made in the landscape of MS therapy with the emergence of novel disease-modifying therapies (DMTs). These therapies primarily act by modulating immune responses, either by controlling immune cells overactivity or by preventing their infiltration into the CNS, subsequently reducing the frequency of relapse and disease severity (Filippi et al. 2022; Noyes and Weinstock-Guttman 2013). However, they display limited efficacy in progressive forms of MS driven by inflammatory demyelination and incomplete repair, presenting an unmet therapeutic gap (Harlow et al. 2015; Nyamoya et al. 2017). With the exception of ocrelizumab, which has shown only a modest effect on slowing disease progression, all 16 United States Food and Drug Administration (FDA)-approved MS therapies fail to halt or substantially delay the progressive accumulation of disability, particularly in progressive forms of MS (Hooijmans et al. 2019). Indeed, failure of remyelination represents a clear obstacle in the recovery of MS, arguing the urgent need for strategies that extend beyond immunosuppression to preserve axonal integrity and restore myelin.

**Fig. 1:**
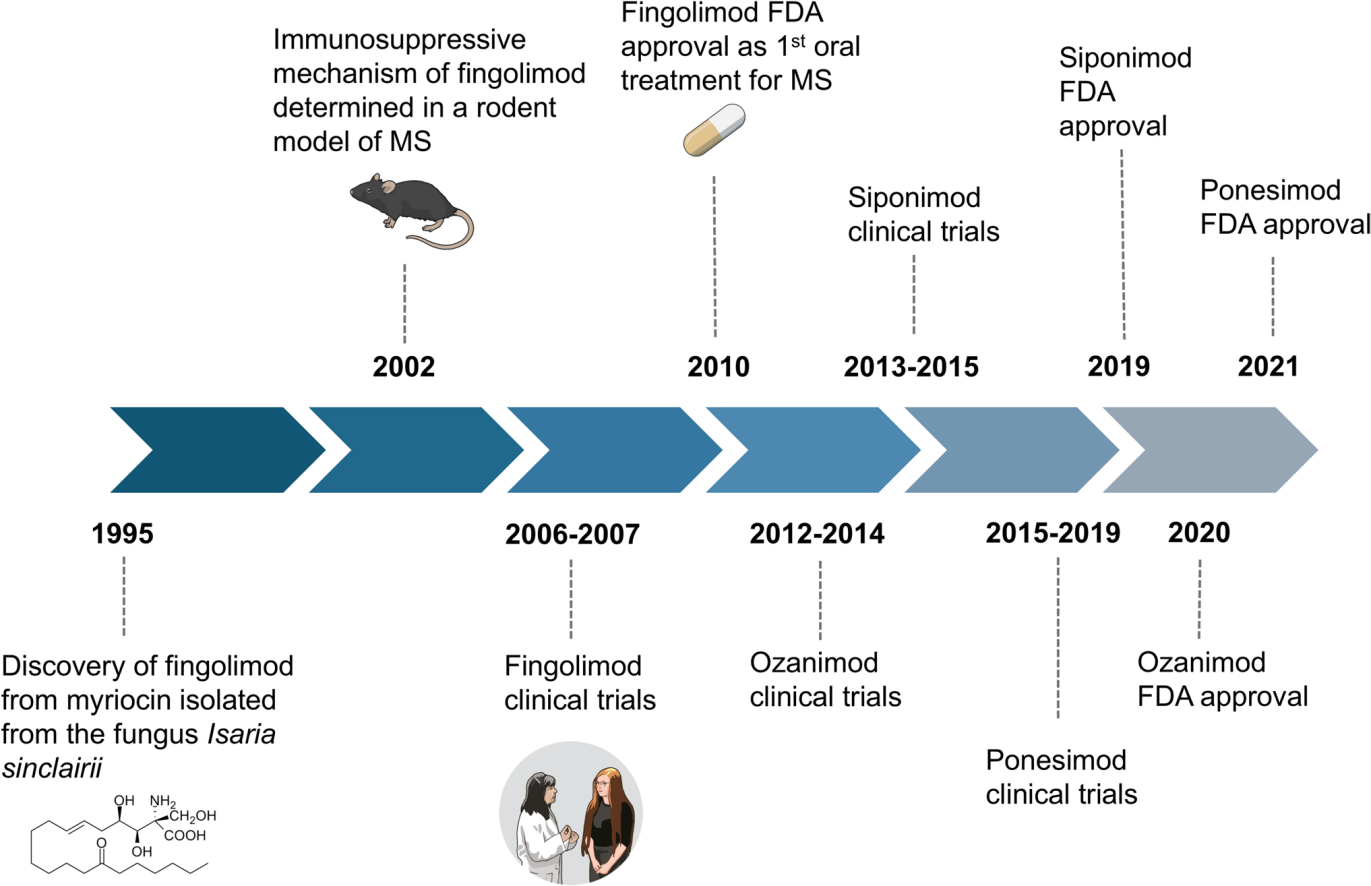
Timeline of key breakthroughs and milestone events in the research of S1P modulators. A schematic showing the timeline of key breakthroughs and milestone events in the research of S1P modulators. Fingolimod was first identified in 1995 as a synthetic derivative of the natural compound myriocin, which was originally isolated from the fungus *Isaria sinclairii* (Park and Im 2017). Its immunosuppressive mechanism was subsequently elucidated using in vitro and in vivo rodent models of MS (Brinkmann et al. 2002). Clinical trials of fingolimod were conducted between 2006 and 2007 (Kappos et al. 2010), leading to its FDA approval in 2010 for the treatment of relapsing forms of MS (Coyle et al. 2024). Following this approval, more receptor-selective S1P modulators targeting S1PR1 and/or S1PR5, including siponimod, ozanimod, and ponesimod, underwent clinical evaluation (Kappos et al. 2018, 2021; Cohen et al. 2019) and were subsequently approved by the FDA in 2019, 2020, and 2021, respectively (Coyle et al. 2024). Figure prepared in Microsoft PowerPoint (available at www.microsoft.com)

Among emerging therapeutic targets, sphingosine 1-phosphate (S1P) has gained substantial attention due to its dual role in immunomodulation and oligodendrocyte lineage biology. S1P exerts its cellular effects through sphingosine 1-phosphate receptors (S1PRs)—a family of five G protein-coupled receptors (S1PR1-S1PR5) (Spiegel and Milstien 2003; Binish and Xiao 2025; Cartier and Hla 2019). Among them, S1PR1 is ubiquitously expressed across multiple cell types, including lymphocytes, where it plays a central role in immunomodulation and therefore represents a key therapeutic target in MS (McGinley and Cohen 2021; Chen et al. 2022). Within the CNS, S1PR1 is expressed by neurons, astrocytes, and microglia, whereas S1PR5 is abundantly and selectively expressed by oligodendrocyte lineage cells, particularly within cortical white matter tracts (Coelho et al. 2010; Bravo et al. 2022). Fingolimod, the first S1PR modulator and oral therapeutic approved for MS (Fig. 1), demonstrated substantial clinical benefit through immunomodulatory mechanisms, but its direct effect on oligodendrocyte lineage cells in vivo remains uncertain. More recently, selective S1PR1/5 modulators such as siponimod, ozanimod, and ponesimod have been developed, raising the possibility that receptor-specific signalling may differentially influence remyelination (Coyle et al. 2024; Roggeri et al. 2020).

Since the approval of fingolimod for relapsing–remitting MS (RRMS) in 2010 (Fig. 3A), increasing preclinical studies have investigated the potential of S1P-based therapeutics not only in preventing demyelination but also influencing olidogdendroglial dynamics and remyelination in vivo. Despite these findings, however, the distinction between preventing demyelination, preserving OLGs, and actively promoting new myelin formation remains unclear. Furthermore, despite extensive clinical use and a growing body of preclinical literature, the extent to which S1P-based therapies promote myelin repair remains unclear and has not been systematically synthesised across mammalian models of demyelination. This systematic review, therefore, aims to evaluate the evidence of remyelination concerning S1P-based therapeutics in in vivo mammalian models of CNS demyelination. Specifically, it examines (i) the efficacy of these treatments in limiting demyelination, (ii) their effects on oligodendroglial dynamics within demyelinated lesions and (iii) their capacity to enhance remyelination as assessed through biochemical, histological, or ultrastructural outcomes. This systematic review provides a comprehensive evaluation, determining the status of current S1P-based therapeutics in regulating myelin repair and identifying key gaps for further research.

## Methods

### Study Design

This systematic review was conducted using the Preferred Reporting Items for Systematic Reviews and Meta-Analyses (PRISMA) guidelines (Page et al. 2021). The methodology was defined prior to conducting the review, and no deviations from the planned approach were made.

### Search Strategy and Study Selection

A systematic literature search was performed on three databases: Web of Science, PubMed, and Scopus to identify all studies that have been published up to the research date (2 July 2025), focusing on the following areas of interest: “remyelination” and “sphingosine-1-phosphate” and “in vivo”. After the selection of appropriate keywords, the search was performed within the “Title and Abstract” in PubMed, “Title, Abstract, and Keyword” in Scopus, and within “Topic” in Web of Science. The publication language was limited to English, and the following advanced searches for each database are reported:

- **Web of Science search:** TS = ("remyelination" OR "myelin repair" OR "myelin" OR "oligodendrocyte") AND TS = (“Sphingosine-1-phosphate" OR "S1P receptor" OR "S1PR" OR "fingolimod" OR "siponimod" OR "ozanimod" OR "ponesimod" OR "etrasimod”) AND TS = ("animal model" OR "mouse model" OR "rodent" OR "in vivo" OR "EAE" OR "cuprizone" OR "lysolecithin" OR "LPS")
- **Pubmed search:** ("remyelination" OR "myelin repair" OR "myelin" OR "oligodendrocyte") AND ( "Sphingosine-1-phosphate" OR "S1P receptor" OR "S1PR" OR "fingolimod" OR "siponimod" OR "ozanimod" OR "ponesimod" OR "etrasimod") AND ("animal model" OR "mouse model" OR "rodent" OR "in vivo" OR "EAE" OR "cuprizone" OR "lysolecithin" OR “LPS”) NOT "review" NOT “comment” NOT “editorial”
- **Scopus search:** TITLE-ABS-KEY ("remyelination" OR "myelin repair" OR "myelin" OR "oligodendrocyte") AND TITLE-ABS-KEY ( "Sphingosine-1-phosphate" OR "S1P receptor" OR "S1PR" OR "fingolimod" OR "siponimod" OR "ozanimod" OR "ponesimod" OR "etrasimod") AND TITLE-ABS-KEY ( "animal model" OR "mouse model" OR "rodent" OR "in vivo" OR "EAE" OR "cuprizone" OR "lysolecithin" OR "LPS") AND NOT "review" AND NOT "comment" AND NOT "editorial"

### Inclusion and Exclusion Criteria

The search strategy was structured using the PICOS (population, intervention, comparator, outcome, study) model, which guided the inclusion criteria.

- **Population**: Animals subjected to CNS demyelination via established experimental models (e.g. cuprizone, lysolecithin, EAE, LPS).
- **Intervention**: Treatment with S1P-based therapeutics (e.g., fingolimod, siponimod, ozanimod, ponesimod, estrasimod).
- **Comparator**: Demyelinated animals receiving no treatment, vehicle, or placebo treatment.
- **Outcome**: Quantitative or qualitative measures of CNS remyelination (e.g., histological assessment of myelin density, oligodendrocyte maturation, myelin protein expression).
- **Study**: In vivo preclinical studies using mammalian models (mouse, rat, etc.)

Only articles that report the effects of S1P-based therapeutics on myelin or oligodendrocyte lineage cells (OLGs/OPCs) were included. This included studies in which S1P-based therapeutics were used to validate a novel compound or drug delivery model, provided that the effects of the S1P-based therapeutic alone were reported and could be extracted for this systematic review. All human studies or in vitro, ex vivo, and in silico studies were excluded. Studies that used a PNS demyelination model were also excluded. Additionally, studies that do not report the dosage of the S1P-based therapeutic were excluded. Finally, articles examining S1P-based therapeutics in in vivo CNS demyelination models were excluded if they did not assess at least one of the following outcomes:

- On preventing myelin damage/demyelination
- On promoting myelin repair/remyelination
- On OLG or OPC survival, differentiation, and maturation

**Fig. 2:**
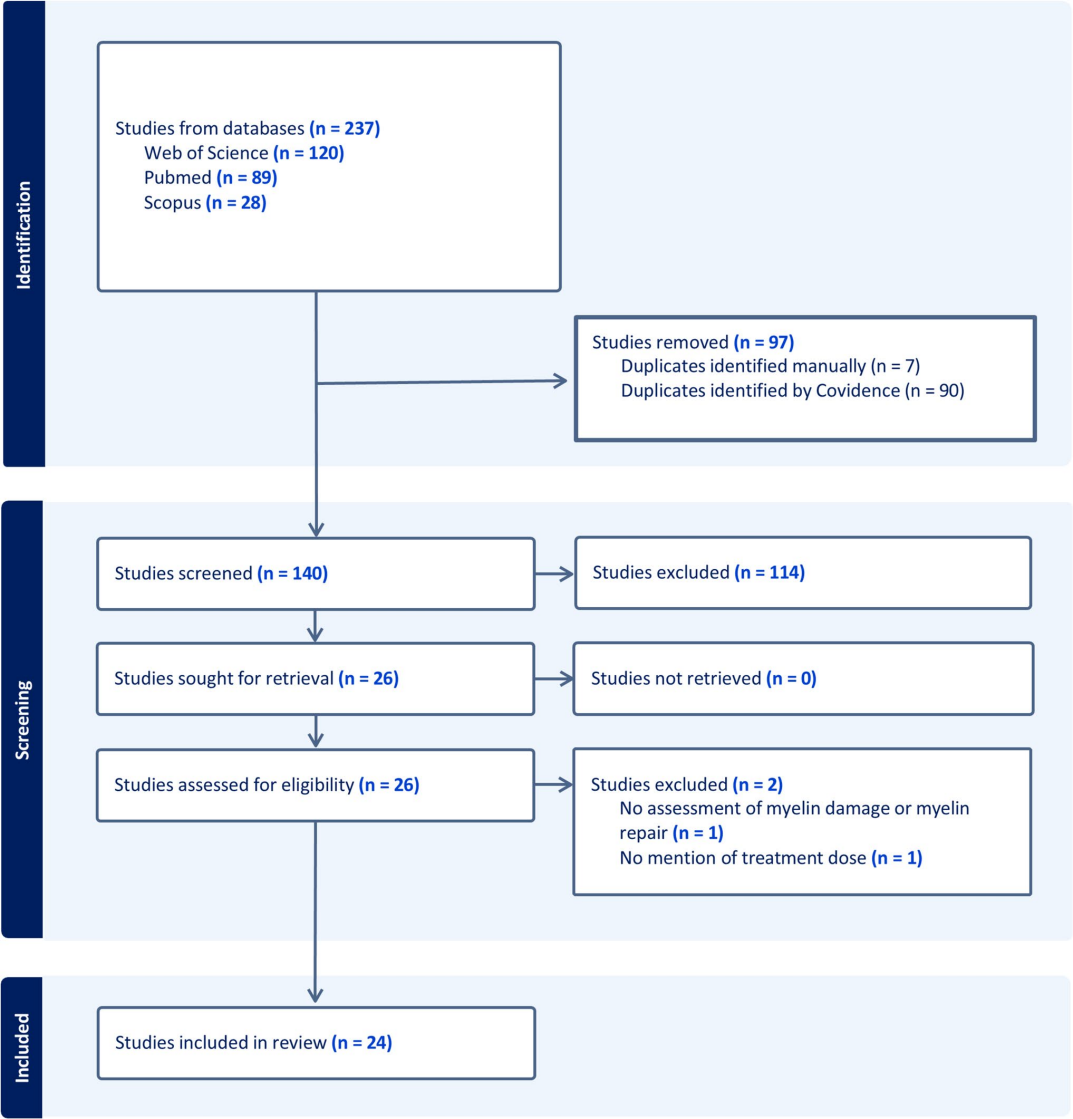
A PRISMA flow diagram showing article selection for the systematic review. Out of 237 studies from three databases, 24 were eligible for inclusion in this systematic review. Flow diagram created by Covidence

Using the Covidence software (available at www.covidence.org), two reviewers independently assessed the titles and abstracts of the identified studies to determine their relevance. Studies that passed the initial screening underwent full-text review using the same process. Any conflicts arising during the screening process were resolved by a third reviewer. This approach allowed enabled the systematic exclusion of studies that did not meet the inclusion criteria and ensured the comprehensive evaluation of study eligibilities for this systematic review. The outcomes of the study selection process are reported in Fig. 2.

### Data Extraction

A standardised data extraction form was developed and pilot-tested to enable any minor adjustments to be made to the template if required. The primary data were extracted by a single reviewer using a custom-designed template to capture all relevant and necessary information from the included studies, which was subsequently reviewed by an independent reviewer. Extracted data were summarised in the tables and included the following information:

- Characteristics of the S1P-based therapeutics, including molecular weight, receptor target(s), evidence of molecular binding to S1PRs, and BBB permeability (Table 1).
- Description of the animal models used in each study, including their characteristics, method of induction, strengths, and limitations (Table 2).
- Efficacy of S1P-based therapeutics in preventing demyelination (Table 3).
- Efficacy of S1P-based therapeutics in promoting remyelination (Table 4).

Due to the inherent heterogeneity of preclinical animal studies, particularly in treatment paradigms, studies were categorised as assessing prevention of demyelination or promoting remyelination based primarily on the timing of intervention and the presence or absence of a defined recovery phase. Studies were classified as assessing prevention of demyelination if they: (i) administered treatment during an ongoing demyelinating phase without a defined recovery period (e.g., continuous CPZ exposure without a subsequent recovery period or progressive EAE models), or (ii) initiated treatment prophylactically prior to the induction of the demyelinating insult. Studies were classified as assessing promotion of remyelination if they included a distinct demyelination phase followed by a recovery period during which treatment effects were evaluated (e.g., CPZ withdrawal paradigms or LPC-induced focal demyelination with tissue collection at defined post-lesion recovery timepoints).

### Risk of Bias Assessment

**Fig. 3:**
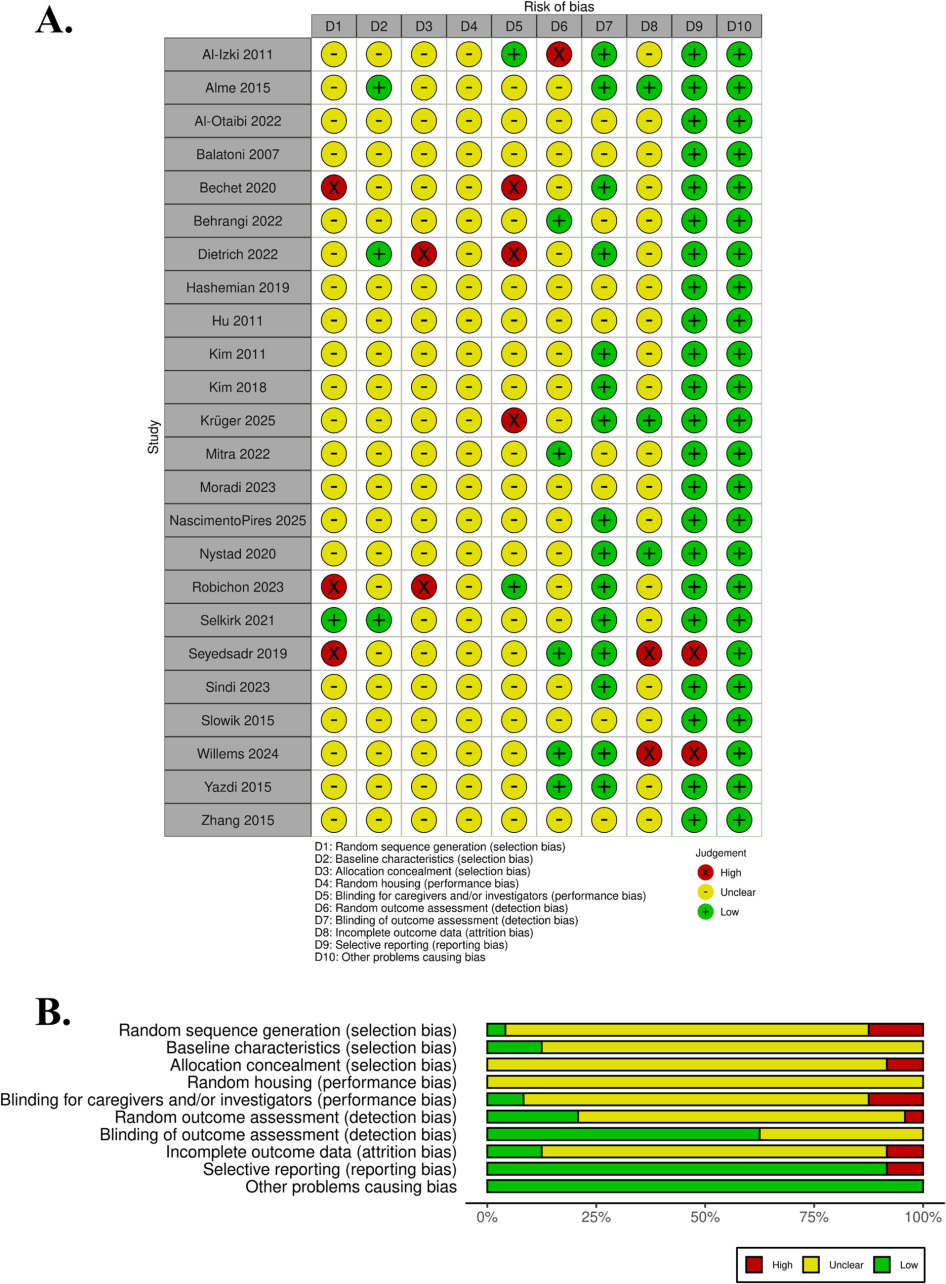
SYRCLE risk of bias assessment of selected articles. All 24 studies were reviewed and assessed using the following criteria: D1: Random sequence generation; D2: Baseline characteristics; D3: Allocation concealment; D4: Random housing; D5: Blinding for caregivers and/or investigators; D6: Random outcome assessment; D7: Blinding of outcome assessment; D8: Incomplete outcome data; D9: Selective reporting; and D10: Other sources of bias. Red: High bias; Yellow: Unclear bias; Green: Low bias (Hooijmans et al. 2014). Both the **A** “stop-light” figure and **B** summary graph were created using the *robvis* Shiny web application (available at shinyapps.io/robvis) (McGuinness and Higgins 2020)

To assess the risk of bias and methodological quality of the included studies, the Systematic Review Centre for Laboratory Animal Experimentation (SYRCLE) risk of bias assessment tool was used. This tool is adapted from the Cochrane risk of bias framework and has been specifically designed to address sources of bias that can be present in animal studies (Hooijmans et al. 2014). The SYRCLE assessment contains ten domains: random sequence generation, baseline characteristics, allocation concealment, random housing, blinding for caregivers and/or investigators, random outcome assessment, blinding of outcome assessment, incomplete outcome data, selective reporting, and other sources of bias. The results of the risk of bias assessment are presented in Fig. 3.

## Results

### Study Selection

A total of 237 studies were initially identified through database searches. After removing 97 duplicates, 140 studies progressed to title and abstract screening. Following the screen, 114 studies were excluded, leaving 26 studies for full-text assessment. Of these, two additional studies were excluded, resulting in a final inclusion of 24 studies in this systematic review. Any conflicts that arose between the two primary reviewers during the screening process were resolved by the third reviewer, ensuring a consistent and thorough selection process.

### Risk of Bias and Quality Assessment

All 24 included studies were critically appraised using the SYRCLE risk of bias assessment tool. Most studies were judged to have an unclear risk of bias across several criteria, such as random sequence generation, baseline characteristics, allocation concealment, random housing, blinding for caregivers and/or investigators, random outcome assessment, and incomplete outcome data. In contrast, most studies demonstrated a low risk of bias for blinding of outcome assessment and selective reporting.

### Characteristics of S1P Modulators and Experimental Models

**Fig. 4:**
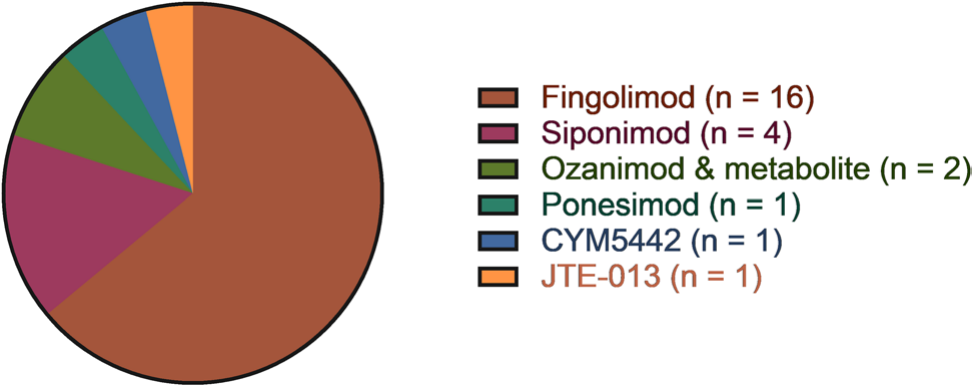
Distribution of research articles on S1P modulators. Of 24 articles, fingolimod (FTY720) was studied most frequently, with 16 studies, followed by siponimod with 4 studies. One study was on ozanimod, RP-101074 (ozanimod metabolite), ponesimod, CYM5442, and JTE-013. One study investigated both the effect of Fingolimod as well as CYM5442 (Kim et al. 2018). Figure prepared in GraphPad Prism (available at www.graphpad.com)

All 24 studies investigated S1PR modulators; however, no studies identified in the screening process examined the modulation of S1P metabolic pathways, such as inhibition of sphingosine kinases (SphK1/2) or sphingosine-1-phosphate lyase (SPL). The characteristics of the S1P-based modulators, and the number of studies investigating each compound are summarised in Table 1 and Fig. 4. Out of the seven compounds identified through the systematic screening, four have been approved by the FDA for the treatment of relapsing forms of MS, including clinically isolated syndrome (CIS), RRMS, and active secondary progressive MS (SPMS). These compounds are fingolimod (approved in 2010), siponimod (approved in 2019), ozanimod (approved in 2020), and ponesimod (approved in 2021) (Table 1 and Fig. 1) (Coyle et al. 2024). The remaining three compounds, RP-101074, CYM5442, and JTE-013 are research tools used in preclinical studies and have not yet progressed to clinical trial stages (Table 1). While all other modulators are S1PR agonists, JTE-013 is unique as a functional antagonist of S1PR2 (Table 1) (Seyedsadr et al. 2019). The characteristics of the animal models used to study demyelination and remyelination are outlined in Table 2. 

**Table 1:** Compound (S1PR modulator) characteristics

Name of modulator	Molecular weight	Targeted S1PR	Molecular binding evidence	BBB permeability
FTY720 (Fingolimod/Gilenya)	307.5 g/mol CID: 107970	S1PR1, S1PR3, S1PR4, S1PR5	S1PR1 (EC_50_ = 0.3 nM) S1PR3 (EC_50_ = 0.9 nM) S1PR4 (EC_50_ = 345 nM) S1PR5 (EC_50_ = 0.50 nM) (Scott et al. 2016)	Brain:blood ratio of 14:1 in Lewis rats after 13 days treatment and 27:1 in Dark Agouti (DA) rats after 23-days treatment of 0.3 mg/kg FTY720. Blood and brain samples taken 24 h after injection. Autoradiography have shown that [^14^C]FTY720 is lipophilic and distributes into the parenchyma of rat CNS following a single oral dose (Foster et al. 2007)
Siponimod (Mayzent)	516.6 g/mol CID: 44599207	S1PR1, S1PR5	S1PR1 (EC_50_ = 0.39 nM) S1PR5 (EC_50_ = 0.38 nM) (Scott et al. 2016)	Brain:blood ratio approaching 6:1 in C57BL/6 mice at various doses ranging from 0.1 to 30 mg/kg of food. Autoradiography showed that [^14^C]siponimod readily penetrated rat CNS, with particularly high uptake in white matter regions (cerebellum, corpus callosum, medulla oblongata) and lower levels in areas such as the olfactory bulb (Bigaud et al. 2021)
Ozanimod (Zeposia)	404.5 g/mol CID: 52938427	S1PR1, S1PR5	S1PR1 (EC_50_ = 0.41 nM) S1PR5 (EC_50_ = 11 nM) (Scott et al. 2016)	Brain:blood ratio of 10:1 in C57BL/6 mice at 1 mg/kg dose, and brain:blood ratio of 16:1 in Sprague–Dawley rats at 0.5 mg/kg (Scott et al. 2016)
RP-101074*	360.4 g/mol (MW referenced from RP-101075; CID: 52938426)	S1PR1, S1PR5	S1PR1 (EC_50_ = 0.35 nM) S1PR5 (EC_50_ = 4.5 nM) RP-101074 data not available, data shown are for RP-101075 (Surapaneni et al. 2021)	RP-101074 BBB permeability data not reported. RP-101075 has a brain:blood ratio of 31:1 (species and dose not specified) (Scott et al. 2013)
Ponesimod (Ponvory)	461.0 g/mol CID: 11363176	S1PR1, S1PR5	S1PR1 (EC_50_ = 5.7 nM) S1PR5 (EC_50_ = 11 nM) (Scott et al. 2016)	According to preclinical and clinical studies, ponesimod does cross the BBB or at least exerts effects within the CNS. However, no quantitative brain:blood ratio or neuroimaging evidence has been disclosed
CYM5442	409.5 g/mol CID: 25110406	S1PR1	S1PR1 (EC_50_ = 1.4 nM) (Gonzalez-Cabrera et al. 2008)	Brain:plasma ratio of 35:1 in C57BL/6J mice 24 h after 10mg/kg CYM5442 injection (Gonzalez-Cabrera et al. 2012)
JTE-013	408.3 g/mol CID: 10223146	S1PR2	S1PR2 (IC_50_ = 17 nM) (Osada et al. 2002)	Similar to ponesimod, no quantitative brain:blood ratio or neuroimaging evidence for JTE-013 has been disclosed

Summary of the characteristics of identified S1P-based therapeutics. Columns include the following: modulator name, molecular weight—data retrieved from the PubChem Compound Database, National Library of Medicine, Maryland, USA (Kim et al. 2025), targeted S1PR, molecular binding evidence to human S1PRs, and evidence of BBB permeability via brain:blood ratio and imaging evidence. *: RP-101074 molecular weight, molecular binding evidence, and BBB permeability data not available, therefore, the data shown are for RP-101075, another ozanimod metabolite. RP-101074 is the R-isomer of this metabolite

*CID* PubChem Compound Identifier, *EC*_*50*_ half-maximal effective concentration, *IC*_*50*_ half-maximal inhibitory concentration

**Table 2:** Animal models used to study de- and remyelination

Animal model	Characteristics and induction	Strengths	Limitations
EAE	Autoimmune-driven demyelination induced by immunisation with myelin antigens in complete Freund’s adjuvant containing pertussis toxin (Constantinescu et al. 2011)	Mimics the pathological features and disease course of MS Robust immune-driven demyelination Well-established model (Constantinescu et al. 2011)	Does not represent progressive MS No clear remyelination/recovery phase (Ransohoff 2012) Demyelination mostly confined to the spinal cord with few brain lesions Pathological characteristics dependent on species and myelin epitope (Palumbo and Pellegrini 2017)
CPZ	Toxin model causing oligodendrocyte apoptosis and demyelination by dietary administration of CPZ (0.2–0.3%) in chow (Ransohoff 2012)	Well-established model with clear demyelination-remyelination timeline (Praet et al. 2014) Minimal peripheral immune involvement (Zhan et al. 2020)	Does not fully recapitulate MS autoimmune pathology Mechanism behind OLG degeneration still poorly understood (Kipp 2024) Region-specific demyelination (Zhan et al. 2020)
CPZ + EAE	A combination of toxin-induced oligodendrocyte loss with autoimmune infiltration by feeding mice with CPZ chow for 3 weeks, followed by normal chow for 2 weeks, and EAE induction (Rüther et al. 2017)	Better reflects MS pathology by combining neurodegeneration and inflammation, allowing the study of remyelination under active inflammatory conditions (Scheld et al. 2016)	Technically demanding Animal welfare concern Not a well-established model yet
EAEON	Autoimmune-driven demyelination of the optic nerve, causing optic neuritis and visual deficits. Mode of induction is similar to EAE (Kezuka et al. 2011)	Useful for studying optic neuritis and visual pathways	Limited to optic nerve If not isolated, can occur with systemic EAE
Twitcher mice (Krabbe’s disease model)	Transgenic mouse model with mutation in the galactocerebrosidase (GALC) gene, leading to psychosine accumulation and demyelination (Suzuki and Suzuki 1995)	Strong model for congenital demyelination with progressive and severe myelin loss (Suzuki and Taniike 1995)	Rapid disease progression—mice have short lifespan (Suzuki and Suzuki 1995)
LPC	Focal injection of lysolecithin (LPC) into regions of the CNS such as the spinal cord, optic nerve, corpus callosum, causing localised demyelination (Plemel et al. 2018)	Fast, localised, and reproducible demyelination Spontaneous remyelination one week after injection (Blakemore and Franklin 2008) Can target specific locations of the CNS	Does not represent widespread demyelination like in MS Invasive injection (Hooijmans et al. 2019)
Light-induced photoreceptor loss (LI-PRL)	Retinal and optic nerve degeneration is induced by exposure to high-intensity light (Dietrich et al. 2020)	Useful to study retinal and optic nerve degeneration independent of peripheral immune infiltration Non-invasive method of induction (Sindi et al. 2023)	Does not represent widespread demyelination and lacks systematic immune involvements No direct link to MS (Dietrich et al. 2020)
Cuprizone/rapamycin demyelination	A combination of CPZ treatment and rapamycin, an inhibitor of the mTOR pathway, providing a more complete demyelination by inhibiting spontaneous remyelination (Yamate-Morgan et al. 2019)	Enhances the severity and completeness of demyelination (Sachs et al. 2014)	Inhibition of mTOR pathways affect other glia and neurons, making interpretation difficult (Yamate-Morgan et al. 2019)

Summary of the characteristics of animal models to study de- and remyelination. Columns include the following: name of animal model, characteristics of the model and mode of induction, strengths, and limitations

### Efficacy of S1P Modulator on Myelination and Oligodendrocytes

The studies employed a wide range of methodological approaches to assess demyelination and remyelination. These included myelin-specific histological stains such as Luxol Fast Blue (LFB), Black-Gold II, toluidine blue, and Sudan Black, as well as immunohistochemistry (IHC), transmission electron microscopy (TEM), Western blotting (WB), polymerase chain reaction (PCR), enzyme-linked immunosorbent assay (ELISA), and magnetic resonance imaging (MRI). However, several studies reported changes in myelin or oligodendrocyte lineage markers without incorporating a clearly defined remyelination phase in their experimental design, making it difficult to distinguish myelin preservation from true remyelination. As a result, studies were grouped according to the predefined criteria described in the Methods section. Studies lacking a distinct recovery phase were categorised as assessing prevention of demyelination (Table 3), whereas those including an explicit remyelination phase were categorised as assessing remyelination (Table 4).

**Table 3:** Efficacy of S1PR modulators in preventing demyelination

	Author	Model and species	Treatment paradigm	Dosage	Sex and sample size	Demyelination outcomes
Fingolimod (FTY720)	Al-Izki et al. (2011)	EAE Biozzi antibody-high mice	Fingolimod started from EAE induction (day 0), at first relapse (day 10), or 1-month post-immunological tolerance (secondary progressive phase) until endpoint at 89 dpi	1mg/kg or 3 mg/kg. Daily oral gavage	Both male and female mice included n = 8–9 per treatment group	No significant difference in toluidine blue staining between FTY720-treated and vehicle groups
	Balatoni et al. (2007)	EAE. Dark agouti (DA) rats	Prophylactic treatment: FTY720 treatment started at time of EAE induction and continued for 2 to 3 weeks. Therapeutic treatment: FTY720 treatment initiated on day 25 after EAE induction and continued for 3 weeks	0.4 mg/kg Daily oral gavage	Female. Sample size not explicitly stated, n = 12 per treatment group	Prophylactic treatment: FTY720 prevented detectable demyelination or lesions in the brain & limited SC demyelination Therapeutic treatment: FTY720 significantly reduced SC demyelination (p < 0.05) & eliminated detectable lesions vs controls. No differences in LFB staining at day 53 (no statistical analysis performed)
	Hashemian et al. (2019)	LPC. Wistar rats	LPC injection followed by FTY720 treatment started on the same day and continued for 7 or 14 days	0.3 mg/kg Daily oral gavage	Male, n = 72	Reduced demyelination at d7 and d14 with FTY720 vs vehicle (quantitative fluoromyelin staining). PLP + cells increased with FTY720 (no statistical analysis reported)
	Hu et al. (2011)	LPC. Sprague–Dawley rats	LPC injection followed by FTY720 treatment started on 2 or 3 dpi and continued for 7 days	Either daily 1 mg/kg oral gavage starting 2 days after induction, or daily 2 µL (2 mg/mL) FTY720 injection into LPC lesion starting 3 days after induction	Sex not mentioned. For LFB staining, control n = 5, FTY720 n = 7. TEM n = 3	Local FTY720 injection of LPC rats doubled demyelinated lesion size compared to vehicle (p < 0.05) and induced demyelination in non-LPC rats. Oral FTY720 did not increase lesion volume
	Kim et al. (2011)	CPZ (demyelination phase only). C57BL/6 mice	6 weeks of 0.2% CPZ feeding, FTY720 treatment started on day 1 and continued for 6 weeks	1 mg/kg. Daily oral gavage	Male, n = 5–15 per treatment group	FTY720 attenuated demyelination, increased MBP intensity (p < 0.002) and proportion of myelinated fibers compared to vehicle (p < 0.002). FTY720 increased CC1 + , Nkx2.2 + , and NG2 + /PCNA + cell numbers (p < 0.013, p < 0.04, p < 0.0003)
	Kim et al. (2018)	CPZ (demyelination phase only). C57BL/6 mice	3 weeks of 0.2% CPZ feeding, treatment started on day 3 or day 10 until the end of week 3	1 mg/kg. Daily intraperitoneal (IP) injection	Male, = 3–11 per analysis per group	Early FTY720 treatment at d3 reduced demyelination and prevented OLG death (fewer TUNEL + cells, p < 0.05). CNPase and MAG WB/PCR confirmed OLG protection (p < 0.01 WB, p < 0.001 PCR). Delayed FTY720 treatment at d10 showed no OLG protection (no change in *Mag/Mbp* gene expression vs vehicle)
	Moradi et al. (2023)	CPZ (demyelination phase only). Wistar rats	Treatment with fingolimod initiated 1 week before the induction of CPZ (0.3%) for 4 weeks. Treatment continued until endpoint	0.5 mg/kg. Daily oral gavage	Male, n = 49	FTY720 increased toluidine blue staining, axon diameter, and myelin thickness in CC (TEM, p < 0.05). MBP and Olig2 levels were elevated vs CPZ/vehicle (PCR, p < 0.05) and approached normal controls (ELISA, p < 0.05)
	Nascimento Pires et al. (2025)	CPZ (demyelination phase only). Swiss mice	5 weeks of 0.2% CPZ feeding, treatment started on day 1 until the end of week 5	1 mg/kg. Daily IP injection	Sex not mentioned, n = 6 per group	FTY720 partially restored lipid density in the CC vs CPZ (Sudan black, p < 0.05). CC cytoarchitecture remained disrupted, with reduced chain-like organisation vs control (H&E, p < 0.005). FTY720 preserved myelinated fibers in the 0.75–0.81 g-ratio range, but mean g-ratio, axon area, and fiber area were unchanged vs CPZ. FTY720 did not increase CC1 + cells but statistically decreased NG2 + cells vs CPZ (p < 0.01)
	Nystad et al. (2020)	CPZ (demyelination phase). C57BL/6 mice	6 weeks of 0.2% CPZ feeding. Treatment administered from the beginning of week 5 for 2 weeks	1 mg/kg. Daily oral gavage	Female, n = 48	FTY720 did not ameliorate myelin loss vs vehicle during demyelination (p = 0.38). No differences observed in LFB or PLP scores in SMC or in myelin levels at any time point. FTY720 did not alter NOGO-A + mature OLG density in the CC during demyelination (p = 0.58). In the SMC, mature OLGs did not increase during demyelination (p = 0.23)
	Robichon et al. (2023)	EAE. C57BL/6 mice	EAE induction on day 0, daily treatment started at disease onset (score ≥ 1; 13–18 dpi) until day 40–55	1 mg/kg. Daily oral gavage	Female, n = 18–20 per treatment group	No significant increase in myelination % with FTY720 vs vehicle (Black-Gold II staining). However, FTY720 significantly reduced lesion percentage vs vehicle (p < 0.05)
	Yazdi et al. (2015)	LPC. C57BL/6 mice	Mice were treated with FTY720 for 8 or 12 days, and LPC was injected from day 6 of the treatment period	0.3 or 1 mg/kg. Daily oral gavage	Male, n = 3 per condition (n = 21 total)	At 6 dpi, LFB staining showed a significant reduction in demyelination in animals treated with FTY720 (0.3 or 1 mg/kg; both p < 0.001) compared to the LPC group
	Zhang et al. (2015)	EAE. SJL/J mice	EAE induction on day 0, daily treatment started on the day of EAE onset until day 7 or day 30 post onset	0.15 or 0.3 mg/kg. Daily oral gavage	Female, n = 36	Western blotting of MBP is not significant between FTY720 and vehicle-treated mice at d7. NG2 + OPCs and BrdU + -NG2 + cells increased in CNS and SVZ at d7 (p < 0.01). FTY720 restored myelination after EAE (LFB, d30 p.o., p < 0.05). At d30, MBP protein for FTY720 significantly higher vs controls (p < 0.05). NG2 + OPCs and BrdU^+^-NG2^+^ cells increased in CNS and SVZ (p < 0.05). CNPase^+^ mature OLGs increased across regions of ST/CC/SC (p < 0.01/0.05/0.01). BrdU^+^-CNPase^+^ cells appeared by d30 (p < 0.05)
Siponimod	Behrangi et al. (2022)	CPZ (demyelination phase only) and CPZ + EAE model. C57BL/6 mice	Treatment started on the same day of 0.25% CPZ feeding and continued for 1–3 weeks. Treatment was then discontinued, and CPZ + EAE mice were returned to normal chow for 2 weeks and received MOG immunisation at week 6	0.315 mg/kg, 3.125 mg/kg, or 15.5 mg/kg. Daily oral gavage	Female, n = 10 per treatment group	Siponimod increased LFB grading (p < 0.01) and CC1 + mature OLGs (p < 0.05) vs vehicle. This effect was dose-dependent, 0.315mg/kg showed greater demyelination (LFB/PLP) than other doses. Siponimod reduced CPZ-induced increase in Olig2 + /PCNA + OPCs in WT but not S1PR5-KO mice, suggesting OLG protection rather than myelin regeneration
	Dietrich et al. (2022)	EAEON. C57BL/6 mice	EAEON was inducted and treatment started on the same day (prophylactic), or at 14 or 30 dpi (therapeutic). Treatment continued until either 21, 35, or 90 dpi	2 or 6 mg/kg daily Siponimod intake via chow	Female, n = 48 (n = 6 per group)	Prophylactic siponimod reduced optic nerve MBP loss vs vehicle (2 mg/kg p < 0.001; 6 mg/kg p < 0.01). Late treatment (d14) was beneficial at 2 mg/kg (p < 0.05), while d30 showed no effect on myelin loss. Therapeutic siponimod (d14) improved Olig2 + cell survival (2 mg/kg p < 0.01; 6 mg/kg p < 0.05), with no change in PDGFRα + cells
	Krueger et al. (2025)	CPZ (demyelination phase only). C57BL/6 mice	4 or 7 weeks of 0.2–0.55% CPZ feeding. 4-week CPZ group received treatment from day 1, and 7-week CPZ group received treatment from week 5 to endpoint	6.25 mg/kg BW. Daily oral gavage	Male, n = 5–8 per group	Siponimod partially reduced demyelination (LFB/PAS, anti-MAG, p < 0.05). Siponimod treatment from week 5 increased MBP and MAG staining (p = 0.0059, p = 0.0093) and raised Olig2 + cell density (p = 0.0869). Treatment from day 1 reduced Olig2 + /Ki67 + OPCs (p = 0.02) but did not significantly increase Olig2 + cell density (p = 0.0869)
CYM5442	Kim et al. (2018)	CPZ (demyelination phase only). C57BL6 mice	5 weeks of 0.2% CPZ feeding, treatment started from 3rd day of CPZ to endpoint	10 mg/kg, daily IP injection	Male, n = 3–8 per analysis per group	CYM5442 reduced demyelination vs vehicle (Sudan Black, p < 0.05). Western blotting for CNPase and MAG at 3 weeks confirmed OLG protection and reduced demyelination (p < 0.05)
Ozanimod	Selkirk et al. (2021)	EAE. C57BL/6 mice	EAE was induced and treatment started on the same day and continued for 14 days	0.05, 0.2, or 1 mg/kg. Daily oral gavage	Female, n = 36 (n = 12 per group)	All ozanimod doses have significantly lower demyelination score vs vehicle (H&E, p < 0.001)
JTE-013	Seyedsadr et al. (2019)	EAE and LPC models. C57BL/6 mice	EAE was induced and treatment started on day 1 p.o. and continued until endpoint (d18). LPC injection followed by treatment initiated on the same day and continued until day of sacrifice at 3, 7, 14 or 16 dpi	EAE: 30 mg/kg. Daily IP injection LPC: 15 mg/kg. Twice daily IP injection	EAE: Male, n = 3–4 per group LPC: Male, n = 13 per group	EAE: JTE-013 reduced demyelinated lesion areas in SC vs vehicle (MBP staining, p < 0.05). EdU + /CC1 + newly differentiated OLGs were higher in JTE-013 mice, but not significantly LPC: At 3 dpi, MBP staining showed no significant difference in demyelination between vehicle and JTE-013, in either the spinal lesion or optic chiasm
RP-101074	Sindi et al. (2023)	LI-PRL. C57BL/6 mice	LI-PRL induction followed by RP-101074 treatment initiated on the same day and continued for 5 weeks	1 mg/kg. Daily oral gavage	Female, n = 5–6 per treatment group	Prophylactic RP-101074 significantly protected myelin (p < 0.0018), increased Sox2^+^ cells (p < 0.0001), and upregulated NG2 (p = 0.0005) and PDGFRα (p = 0.0001) vs vehicle, indicating enhanced OPC activation and myelin preservation

Summary of the efficacy of S1PR modulators on demyelination in mammalian in vivo models. Columns include the following: modulator type; author and year of publication; in vivo model and species; treatment window; dosage; sex and sample size; and demyelination outcome

*CC* corpus callosum, *dpi* days post-induction, *H&E* hematoxylin and eosin, *KO* knock-out, *MAG* myelin-associated glycoprotein, *MBP* myelin basic protein, *PAS* Periodic acid–Schiff, *PLP* proteolipid protein, *p.o.* post-onset, *SC* spinal cord, *SMC* secondary motor cortex, *ST* striatum, *SVZ* subventricular zone, *WT* wild-type

**Table 4:** Efficacy of S1PR modulators on promoting remyelination

	Author	Model and species	Treatment paradigm	Dosage and delivery	Sex and sample size	Remyelination Outcomes
Fingolimod (FTY720)	Alme et al. (2015)	CPZ with remyelination phase. C57BL/6 mice	6 weeks of 0.2% CPZ feeding followed by normal chow. Treatment started from week 5 and continued for 4 weeks	1 mg/kg. Daily oral gavage	Female, n = 32	No significant differences in remyelination (MBP and PLP staining) and mature OLGs (NOGO-A^+^) compared to vehicle control
	Béchet et al. (2020)	Twitcher mice	FTY720 treatment started on postnatal day 21 and continued until animal endpoint (postnatal day 40 – 45)	1 mg/kg. Fingolimod reconstituted in drinking water daily	Male and female, n = 20 – 33	FTY720 increased MBP expression significantly (IHC, p = 0.04) but did not alter MOG expression (IHC, p = 0.248) or myelin debris levels in wild-type or twitcher mice (p > 0.999). FTY720 significantly increased Olig2 protein expression vs vehicle in cerebellum (p < 0.05)
	Hashemian et al. (2019)	LPC Wistar rats	LPC injection followed by treatment on the same day. Treatment continued for 7 or 14 days	0.3 mg/kg. Daily oral gavage	Male, n = 72	FTY720 significantly increased *MBP* gene levels on d7 and d14 post-lesion vs vehicle. *Olig2* gene expression increased on d7 (p < 0.05), but not on d14. Remyelination outcome was not available at protein or structural level
	Hu et al. (2011)	CPZ + rapamycin with remyelination phase. C57BL/6 mice	4 weeks of 0.3% CPZ + 10 mg/kg rapamycin 5 days/week, followed by normal chow for 2 weeks. Treatment initiated during normal chow phase and continued for 2 weeks	1 mg/kg Daily oral gavage	Sex not mentioned n = 4 each group (IHC, 12 total) n values unclear for TEM	No significant differences in remyelination between FTY720- and control-treated animals via IHC (MBP) and TEM in either model, although FTY720 increased NG2^+^ cells (p < 0.001) in the CPZ model
	Kim et al. (2011)	CPZ with remyelination phase. C57BL/6 mice	6 weeks of 0.2% CPZ feeding followed by normal chow; treatment started from week 4 – 6 of CPZ diet for 4 weeks	0.3–1 mg/kg. Daily oral gavage	Male, n = 5 – 15 per group	No significant differences in remyelination by LFB and IHC (MBP) staining as well as OLG/OPC numbers between FTY720 and vehicle
	Kim et al. (2018)	CPZ with remyelination phase. C57BL/6 mice	5 weeks of 0.2% CPZ feeding followed by normal chow. Treatment administered from the start of week 5 for 2 weeks	10 mg/kg, daily intraperitoneal (IP) injection	Male, n = 3 – 5 per group per analysis	No significant differences in myelin intensity (Oil Red staining), Olig2^+^ cells, or Olig2^+^/CC1^+^ cells in the corpus callosum of FTY720 vs vehicle-treated mice. Similarly, PLP⁻ area, lesion area, and Olig2 cellularity in the cerebellum did not differ between groups
	Mitra et al. (2022)	CPZ with remyelination phase. Sprague–Dawley rats	5 weeks of 0.2% CPZ feeding followed by normal chow. Treatment administered from the start of week 6 for 2 weeks	3 mg/kg, daily IP injection	Female, n = 10 for each treatment group	No significant differences in remyelination by LFB following FTY720 treatment, although it partially restored demyelinated area of the median CC
	Nystad et al. (2020)	CPZ with remyelination phase. C57BL/6 mice	6 weeks of 0.2% CPZ feeding followed by normal chow. Treatment administered from week 5 to week 9	1 mg/kg. Daily oral gavage	Female, n = 48	No significant differences in remyelination via LFB or IHC (PLP) after 1 (p = 1.0; p = 0.96), or 3 weeks (p = 0.40; p = 0.28) of FTY720 vs vehicle. FTY720 exerted no effects on NOGO-A + mature OLG at 1 week or 3 weeks of remyelination in the CC, although increased mature OLGs after 3 weeks of remyelination (p = 0.032) in the SMC, but not after 1 week (p = 0.66)
	Slowik et al. (2015)	CPZ (both acute and chronic) with remyelination phase. C57BL/6 mice	Mice received 0.2% CPZ for 5 weeks (acute) or 12 weeks (chronic), followed by 11 or 28 days of recovery on normal chow. Treatment administered during the recovery phase for 11 or 28 days	0.3 mg/kg. Daily oral gavage	Male, n = 72	No significant differences in remyelination (PLP, LFB staining) and Olig2^+^ cells following FTY720 administration in acute and chronic CPZ models
	Yazdi et al. (2015)	LPC C57BL/6 mice	Mice were treated with FTY720 for 8 or 12 days, and LPC was injected from day 6 of the treatment period	0.3 or 1 mg/kg	Male, n = 3 per condition (n = 21 total)	0.3 mg/kg FTY720 increased PLP myelination (p < 0.05) and myelinated axons. Both doses reduced g-ratio (p < 0.001), with 0.3 mg/kg more effective. 0.3 mg/kg increased OLG lineage cells vs vehicle control (p < 0.01) and 1 mg/kg (p < 0.05). BrdU^+^/Olig2^+^ increased with FTY720 treatment (p < 0.001)
Siponimod	Al-Otaibi et al. (2022)	CPZ with remyelination phase Swiss mice (SWR/J)	6 weeks of 0.3% CPZ feeding followed by normal chow. Treatment started from week 5 and continued for 4 weeks (until the end of week 9)	1.5 mg/kg. Daily oral gavage	Male, n = 85	Siponimod significantly increased the percentage of myelinated areas in the CC at early (p < 0.0001) and late (p = 0.0026) remyelination stages vs CPZ (LFB staining)
	Dietrich et al. (2022)	CPZ with remyelination phase C57BL/6 mice	5 weeks of 0.2% CPZ feeding followed by Siponimod or drug-free chow (vehicle) for 2 additional weeks	2 mg/kg Daily intake via chow	Female, n = 21	Siponimod exerted a significant effect on remyelination in the CC compared to vehicle control, as assessed via MRI No significant on remyelination (via LFB myelin staining) and mature oligodendrocytes (GSTπ^+^)
Ozanimod	Selkirk et al. (2021)	CPZ + rapamycin with remyelination phase C57BL/6 mice	6 weeks of 0.3% CPZ feeding with daily treatment and rapamycin injection. Treatment started from day 1 and continued for 18 weeks	5 mg/kg Daily oral gavage	Male, n = 6 – 12 per group	No significant differences in remyelination in the cortex, CC, or hippocampus by IHC (PLP staining) and MRI compared to vehicle control
JTE-013	Seyedsadr et al. (2019)	LPC C57BL/6 mice	LPC induction and treatment were initiated on the same day. Treatment continued until endpoints at 3, 7, 14 or 16 dpi	15 mg/kg. Twice daily IP injection	Male, n = 13 per treatment group	At 16 dpi, JTE-013 showed a 2.09-fold increase in remyelinated axons vs vehicle (Sudan black staining), confirmed by semithin sections (p < 0.05). No effect on myelin thickness (via TEM). JTE-013 exerted no significant effect on OLG proliferation at 7 dpi, but increased the number of newly differentiated oligodendrocytes at 14 dpi and 16 dpi (p < 0.05), indicating potentiated differentiation
Ponesimod	Willems et al. (2024)	CPZ with remyelination phase C57BL/6 mice	6 weeks of 0.3% CPZ feeding followed by normal chow. Treatment initiated 3 days prior to CPZ withdrawal and continued for 10 days	1, 3, or 10 mg/kg Daily oral gavage	Male, n = 9 – 11 per treatment group	Ponesimod increased remyelination in the medial CC at all three doses, assessed via IHC (MBP) and TEM (g-ratios). Cortical myelination increased only at 3 mg/kg (p = 0.0232)

Summary of the efficacy of S1PR modulators on remyelination in mammalian in vivo models. Columns include the following: modulator type; author and year of publication; in vivo model and species; treatment window; dosage; sex and sample size; and remyelination outcome. *CC* corpus callosum, *dpi* days post-induction, *SC* spinal cord, *SMC* secondary motor cortex

The systematic review synthesised 24 mammalian in vivo studies investigating the effects of S1P-based therapeutics on demyelination and remyelination in mammalian models of CNS injury. Of these, 18 studies evaluated the effects of S1PR modulation on the prevention of demyelination, including 12 studies on fingolimod (Kim et al. 2018, 2011; Al-Izki et al. 2011; Balatoni et al. 2007; Hashemian et al. 2019; Hu et al. 2011; Moradi et al. 2023; Nascimento Pires et al. 2025; Nystad et al. 2020; Robichon et al. 2023; Yazdi et al. 2015; Zhang et al. 2015), three on siponimod (Behrangi et al. 2022; Dietrich et al. 2022; Krueger et al. 2025), and one study each on CY5442 (Kim et al. 2018), ozanimod (Selkirk et al. 2021), JTE-013 (Seyedsadr et al. 2019), and RP-101074 (Sindi et al. 2023). Among these studies, 15 reported a reduction in demyelination (Seyedsadr et al. 2019; Kim et al. 2018, 2011; Sindi et al. 2023; Balatoni et al. 2007; Hashemian et al. 2019; Moradi et al. 2023; Nascimento Pires et al. 2025; Robichon et al. 2023; Yazdi et al. 2015; Zhang et al. 2015; Behrangi et al. 2022; Dietrich et al. 2022; Krueger et al. 2025; Selkirk et al. 2021), two reported no significant effect of fingolimod (Al-Izki et al. 2011; Nystad et al. 2020), and one reported increased demyelination following local delivery of fingolimod into LPC lesions (Hu et al. 2011). Across the demyelination-prevention studies, outcomes were assessed using a variety of readouts. Four studies quantified changes in demyelinated lesion number and/or size (Seyedsadr et al. 2019; Balatoni et al. 2007; Hu et al. 2011; Robichon et al. 2023), 16 reported changes in myelin staining intensity (Seyedsadr et al. 2019; Kim et al. 2018, 2011; Sindi et al. 2023; Al-Izki et al. 2011; Hashemian et al. 2019; Moradi et al. 2023; Nascimento Pires et al. 2025; Nystad et al. 2020; Robichon et al. 2023; Yazdi et al. 2015; Zhang et al. 2015; Behrangi et al. 2022; Dietrich et al. 2022; Krueger et al. 2025; Selkirk et al. 2021), and 11 quantified oligodendrocyte lineage cell numbers (Seyedsadr et al. 2019; Kim et al. 2018, 2011; Sindi et al. 2023; Hashemian et al. 2019; Nascimento Pires et al. 2025; Nystad et al. 2020; Zhang et al. 2015; Behrangi et al. 2022; Dietrich et al. 2022; Krueger et al. 2025). Fewer studies assessed biochemical or ultrastructural outcomes, with three reporting change in myelin-associated gene or protein expression (e.g., MBP, MAG, PLP) (Kim et al. 2018; Moradi et al. 2023; Zhang et al. 2015), and only two using TEM analyses (g-ratio, proportion of myelinated axons) (Moradi et al. 2023; Nascimento Pires et al. 2025).

During active demyelination, S1PR modulation consistently altered oligodendrocyte lineage cell dynamics in a manner indicative of enhanced cell preservation and survival. Across models, treatment was associated with increased numbers of mature oligodendrocytes marked by CC1^+^, Nkx2.2^+^ (Kim et al. 2011), and NOGO-A^+^ (Nystad et al. 2020), alongside reductions in TUNEL^+^ apoptotic oligodendrocytes (Kim et al. 2018), suggesting protection from injury-induced cell death. Concurrent expansion of oligodendrocyte precursor populations, reflected by increases in Olig2^+^/PCNA^+^ (Behrangi et al. 2022), Olig2^+^/Ki67^+^ (Krueger et al. 2025), NG2^+^ and BrdU^+^/NG2^+^ cells (Zhang et al. 2015), further indicates an enhancement in the proliferative capacity following demyelination.

A total of 15 studies examined the effects of S1PR modulation on enhancing remyelination, including 10 studies on fingolimod (Kim et al. 2018, 2011; Hashemian et al. 2019; Hu et al. 2011; Nystad et al. 2020; Yazdi et al. 2015; Alme et al. 2015; Béchet et al. 2020; Mitra et al. 2022; Slowik et al. 2015), two on siponimod (Dietrich et al. 2022; Al-Otaibi et al. 2022), and one study each on ozanimod (Selkirk et al. 2021), JTE-013 (Seyedsadr et al. 2019), and ponesimod (Willems et al. 2024). Remyelination outcomes were most commonly assessed using immunohistological myelin staining (14/15 studies) (Seyedsadr et al. 2019; Kim et al. 2018, 2011; Hu et al. 2011; Nystad et al. 2020; Yazdi et al. 2015; Dietrich et al. 2022; Selkirk et al. 2021; Alme et al. 2015; Béchet et al. 2020; Mitra et al. 2022; Slowik et al. 2015; Al-Otaibi et al. 2022; Willems et al. 2024), followed by oligodendrocyte lineage cell quantification (9/15 studies) (Seyedsadr et al. 2019; Kim et al. 2018, 2011; Hu et al. 2011; Nystad et al. 2020; Yazdi et al. 2015; Dietrich et al. 2022; Alme et al. 2015; Slowik et al. 2015). Only two studies reported gene or protein expression analyses (Hashemian et al. 2019; Béchet et al. 2020), four reported TEM assessments (Seyedsadr et al. 2019; Hu et al. 2011; Yazdi et al. 2015; Willems et al. 2024), and two utilised MRI-based measures (Dietrich et al. 2022; Selkirk et al. 2021). Overall, the remyelination outcomes were variable. Among fingolimod studies, only one reported significant remyelination (Yazdi et al. 2015), while eight studies reported no significant enhancement compared to controls or no evidence of remyelination at the structural level (Kim et al. 2018, 2011; Hashemian et al. 2019; Hu et al. 2011; Nystad et al. 2020; Alme et al. 2015; Mitra et al. 2022; Slowik et al. 2015). One study reported mixed findings, with no change in MOG immunostaining but significant in MBP immunostaining and Olig2 protein expression (Béchet et al. 2020). Siponimod showed mixed effects in one study (Dietrich et al. 2022) and a significant remyelinating effect in another (Al-Otaibi et al. 2022). Ozanimod did not significantly enhance remyelination (Selkirk et al. 2021), while JTE-013 increased myelin staining without corresponding improvements in g-ratio measurements (Seyedsadr et al. 2019). Ponesimod demonstrated a significant remyelinating effect; however, this finding was based on a single study (Willems et al. 2024), a limitation shared by several other S1PR modulators in this review.

The heterogeneity of remyelination findings is further corroborated by the changes in oligodendrocyte lineage markers following S1P-based therapeutics. Several studies reported no significant changes in mature OLG markers, including NOGO-A^+^, CC1^+^, or Olig2^+^ cells (Kim et al. 2018; Hu et al. 2011; Alme et al. 2015), following fingolimod treatment, despite preserved lineage populations during earlier demyelinating phases. Nevertheless, some studies have reported the engagement of reparative pathways by promoting OPC differentiation. Increases in NG2^+^ OPCs and BrdU^+^/Olig2^+^ populations were observed following fingolimod treatment (Hu et al. 2011; Yazdi et al. 2015) indicating lineage activation at early and intermediate stages of repair. Modest increases in GST-π^+^ mature oligodendrocytes were also reported following siponimod treatment, although not significant (Dietrich et al. 2022). A pronounced lineage progression was observed following treatment JTE-013, which increased both EdU^+^/PDGFRα^+^ and EdU^+^/CC1^+^ populations (Seyedsadr et al. 2019), and RP-101074, which expanded Sox2^+^ progenitor pools (Sindi et al. 2023).

Fingolimod was the most extensively studied compound (16/24 studies) and consistently prevented and reduced demyelination across EAE, LPC, and CPZ models based on histological evidence. However, in studies explicitly designed to assess remyelination, fingolimod generally failed to enhance myelin thickness, g-ratio, or the degree of myelin staining. The two studies reporting significant remyelination following fingolimod treatment utilised the LPC and EAE models (Yazdi et al. 2015; Zhang et al. 2015), which differ fundamentally from the CPZ model and do not always include a clearly defined remyelination phase. In contrast, newer S1PR1/5-selective modulators such as siponimod and ponesimod demonstrated more consistent evidence of remyelination, reflected by increased myelin staining intensity, enhanced oligodendrocyte lineage marker expression, reduced g-ratio values, and a higher proportion of remyelinated axons (Al-Otaibi et al. 2022; Willems et al. 2024).

**Fig. 5:**
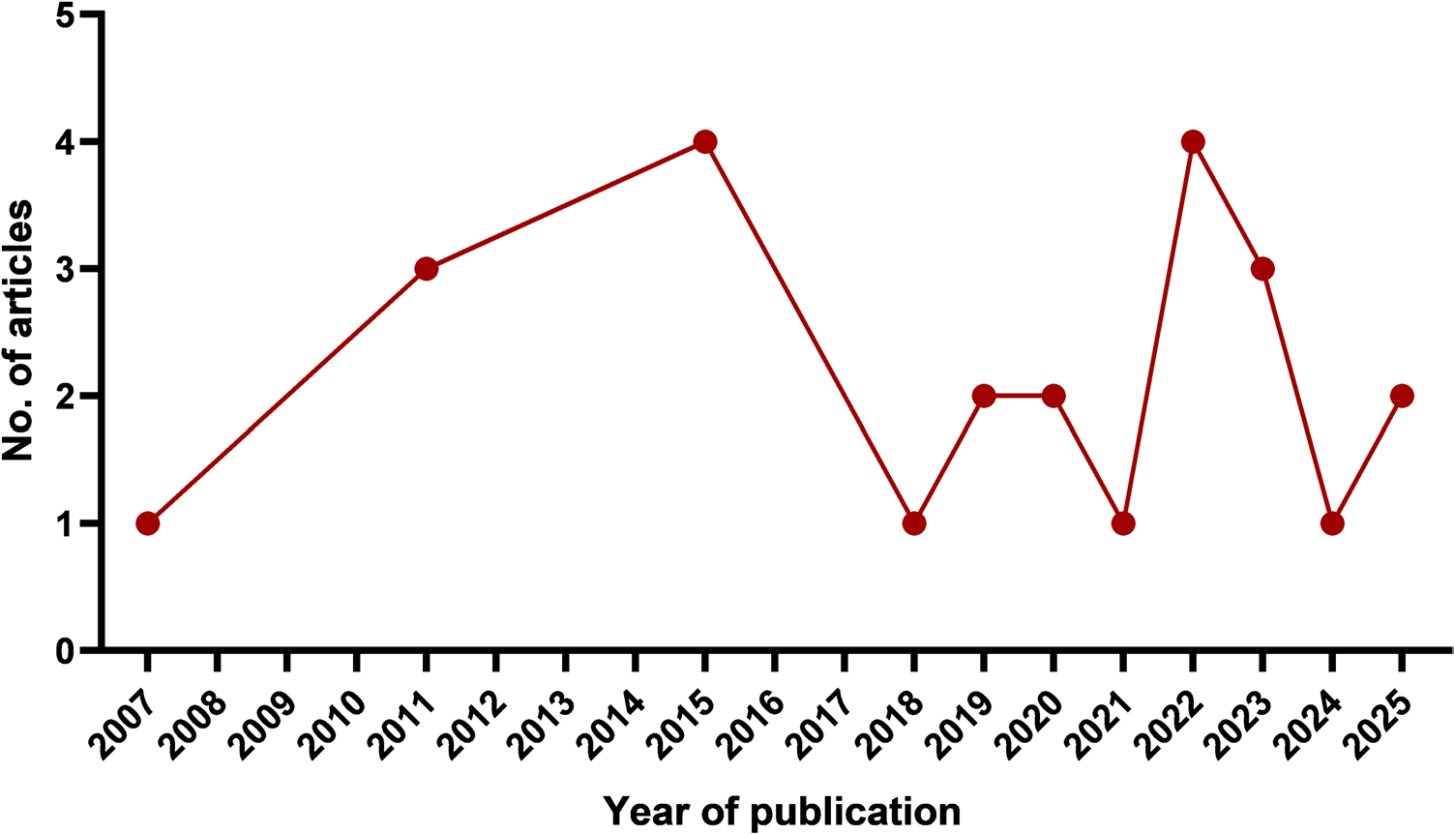
Distribution of S1P modulator research published over time. Line graph showing the number of published articles reporting the effect of S1PR modulation on remyelination in mammalian in vivo models. Figure prepared in GraphPad Prism (available at www.graphpad.com)

Analysis of publication numbers (Fig. 5) reveals that publication activity within this research topic was initially limited, with a single study published in 2007 (Balatoni et al. 2007). Research output increased gradually over the following years, reaching an initial peak of four publications in 2015 (Yazdi et al. 2015; Zhang et al. 2015; Alme et al. 2015; Slowik et al. 2015). This was followed by a decline in activity, with only one study published in 2018 (Kim et al. 2018). A second peak of four publications was observed in 2022 (Behrangi et al. 2022; Dietrich et al. 2022; Mitra et al. 2022; Al-Otaibi et al. 2022), after which output decreased again, although at least one study has been published each year from 2022 onwards.

Between 2007 and 2015, all eight published studies focused exclusively on fingolimod (Balatoni et al. 2007; Hashemian et al. 2019; Hu et al. 2011; Kim et al. 2011; Zhang et al. 2015; Al-Izki et al. 2011; Alme et al. 2015; Slowik et al. 2015). The scope of investigated compounds began to expand afterwards, with CYM5442 studied in 2018 alongside fingolimod (Kim et al. 2018), followed by the investigation of the S1PR2 antagonist JTE-013 in 2019 (Seyedsadr et al. 2019). More selective and newer S1PR1/5 dual modulators were introduced in subsequent years, including ozanimod in 2021 (Selkirk et al. 2021), RP-101074 in 2023 (Sindi et al. 2023), and ponesimod in 2024 (Willems et al. 2024). Siponimod was examined in three studies published in 2022 (Behrangi et al. 2022; Dietrich et al. 2022; Al-Otaibi et al. 2022) and in another study in 2025 (Krueger et al. 2025). Fingolimod remained the most extensively studied compound throughout the entire publication period, with additional studies reported in 2019 (Hashemian et al. 2019), 2020 (Nystad et al. 2020; Béchet et al. 2020), 2022 (Mitra et al. 2022), 2023 (Moradi et al. 2023; Robichon et al. 2023), and 2025 (Nascimento Pires et al. 2025). Overall, these results suggest that research on S1P modulators focusing on understanding remyelination still remains limited.

## Discussion

Overall, the findings of this review reveal a clear distinction between the capacity of S1P-based modulators in preventing demyelination and actively promoting remyelination. Across in vivo models, modulation of S1P signalling consistently limits myelin loss and preserves oligodendroglial populations following demyelination; however, these protective effects do not uniformly translate into enhanced myelin repair. This observation suggests that myelin protection and remyelination represent biologically distinctive processes rather than sequential outcomes of the same therapeutic effect. Consequently, the efficacy of S1P receptor modulation appears to be highly dependent on the disease stage and lesion environment, highlighting the importance of treatment timing, dosage, and model selection when evaluating a compound’s therapeutic potential. However, the SYRCLE risk of bias assessment indicated that most included studies were rated as having an unclear risk of bias across multiple domains (Fig. 3). Although no consistent pattern was observed between risk of bias ratings and the reported effectiveness of S1P modulators in promoting remyelination, the predominance of unclear risk of bias reduces confidence in the overall body of evidence and somewhat limits the strength of conclusions that can be drawn.

### Trends in S1P Modulator Development

As shown in Fig. 5, the initial peak in publications in 2015 likely reflects increased research interest following the approval of fingolimod as the first oral therapy for multiple sclerosis in 2010. During this period, preclinical studies primarily focused on characterising fingolimod’s effects on demyelination, oligodendrocyte lineage dynamics, and early remyelination across toxin-induced and autoimmune models. The second peak observed in 2022 coincided with the emergence of more selective S1PR modulators, including siponimod and ozanimod, within the clinical development pipeline. This shift was accompanied by an expansion in research focus from validating fingolimod to investigating subtype-specific S1PR modulation. These newer compounds preferentially target S1PR1 and S1PR5, receptors implicated in neuroinflammation and oligodendrocyte lineage cell survival, potentially reducing the cardiovascular adverse effects associated with S1PR3 signalling seen with fingolimod’s non-specific receptor modulation (Calabresi et al. 2014; Khatri et al. 2011; Forrest et al. 2004). Together, current research investigating S1P modulators in remyelination heavily focuses on targeting individual S1PRs.

### Efficacy of S1P Modulators on Myelin Protection and Repair

Across in vivo models of demyelination, S1P modulators consistently demonstrate efficacy in preserving myelin integrity during active demyelination, with more than 80% (15/18) of studies reporting a reduction in myelin loss following treatment. These protective effects are most evident in immune-mediated models such as EAE, where modulation of neuroinflammatory processes limits oligodendrocyte injury and subsequent myelin loss. In toxin-based models, including CPZ and LPC, treatment during the demyelination phase frequently reduces lesion severity and preserves myelin-associated markers. However, distinguishing true myelin repair from protection of existing myelin remains challenging, as evidence for effective remyelination was considerably heterogeneous. In many cases, remyelination did not exceed the level of spontaneous repair observed following toxin withdrawal or resolution of inflammation, particularly in fingolimod, where only 1/10 study showed significant enhancement following fingolimod treatment (Yazdi et al. 2015). Interpretation of remyelination outcomes is further constrained by the limited number of studies available for several compounds, including ozanimod, JTE-013, and ponesimod, which prevents definitive conclusions regarding their therapeutic potential.

The interpretation of remyelination outcomes in vivo is complicated due to a range of methodological and biological factors. A major source of variability arises from differences in drug dosage, treatment timing, and therapeutic strategy across studies. In this systematic review, S1P-based therapeutics were administered using both prophylactic and therapeutic paradigms, with some studies initiating treatment before (Moradi et al. 2023; Yazdi et al. 2015) or at the onset of demyelination (Sindi et al. 2023; Nascimento Pires et al. 2025), while others commenced treatment during defined recovery phases to initiate remyelination, most commonly following CPZ withdrawal (Kim et al. 2011; Alme et al. 2015). Although prophylactic paradigms are valuable for studying drug mechanisms, they do not fully reflect the clinical context of demyelinating diseases such as MS, where treatment is typically initiated following diagnosis. This distinction is especially important, as early intervention in patients with undiagnosed or alternative inflammatory CNS conditions may carry an increased risk of adverse effects, further limiting the translational relevance of prophylactic treatment strategies (Ford 2020).

Across the remyelination studies included in this review, enhanced remyelination was often inferred from increases in myelin protein expression or histological staining alone, without concurrent assessment of axonal ensheathment, internode length, or myelin thickness. This limitation was evident in studies such as JTE-013 treatment, where increased myelin staining was not supported by corresponding ultrastructural changes in myelin thickness (Seyedsadr et al. 2019). While these measures can reflect changes in myelination and OLG lineage dynamics, their interpretation is limited in the absence of complementary ultrastructural analysis. TEM therefore remains the gold standard for evaluating remyelination, as it enables the direct assessment of compact, functionally relevant myelin wrapping around axons (Franklin and Ffrench-Constant 2017; Keough and Yong 2013). However, relatively few studies incorporated such analyses, with only four remyelination studies reporting ultrastructural evaluation, contributing to uncertainty in the interpretation of remyelination outcomes. Accordingly, TEM is best used in combination with histological and biochemical analyses, where possible, to provide complementary insights into remyelination at ultrastructural, molecular, and cellular levels. Studies whose conclusions are based solely on histological or myelin staining should therefore be interpreted with greater caution than those employing a comprehensive multimodal approach. Substantial heterogeneity is also introduced by differences in the models of CNS demyelination, animal species and strain, age, and sex. Different in vivo models exhibit markedly different intrinsic remyelination efficiencies. Toxin-induced models such as LPC or CPZ often display robust and rapid remyelination, whereas immune-mediated models such as EAE exhibit incomplete or delayed repair due to persistent inflammation and an unfavourable lesion microenvironment that limits reliable assessment of remyelination. Consequently, therapeutic efficacy observed in one model may not translate directly to another. Several studies also reported remyelination-related outcomes without incorporating a defined remyelination phase from CPZ withdrawal, making it difficult to distinguish enhanced myelin repair from myelin preservation. Additional challenges also involve pharmacokinetics, as many studies did not report the receptor engagement or BBB permeability of S1PR modulators such as ponesimod, RP-101074, and JTE-013, making it difficult to link observed biological effects to specific pharmacological mechanisms. Differences in BBB permeability across models, species, and disease stages can further complicate interpretation (O'Brown et al. 2018). Together, these limitations hinder cross-study comparison and make it difficult to distinguish true pro-remyelinating effects from myelin protection, underscoring the need for more rigorous and standardised experimental designs when evaluating S1PR-targeting therapeutics (Moradi et al. 2023; Krueger et al. 2025). Despite the study heterogeneities, our systematic analysis indicates that current S1P modulators possess efficacy in protecting myelin against a demyelinating insult, but their roles in potentiating myelin formation after injury remain to be determined.

### Efficacy of S1P Modulators on Oligodendroglial Dynamics in Demyelinated Lesions

Across demyelination models, S1P-based therapeutics exert a consistent effect on oligodendroglial lineage preservation and early lineage activation during active demyelination but display limited capacity to drive full lineage progression during recovery. Increases in markers of mature OLG such as CC1, NOGO-A, and Nkx2.2, together with reduced apoptotic TUNEL labelling (Kim et al. 2018, 2011), indicate that S1PR modulation primarily enhances OLG survival and resistance to injury rather than replacement of lost cells during demyelination. The concurrent expansion of OPC populations, reflected by increased Olig2^+^/Ki67^+^, Olig2^+^/PCNA^+^, NG2^+^, and BrdU^+^/NG2^+^ cells, further suggests that S1PR modulation maintains OPC pools during demyelinating phases, which may preserve the cellular substrate required for subsequent myelin repair (Zhang et al. 2015; Behrangi et al. 2022; Krueger et al. 2025).

However, preservation of oligodendroglial lineage cells does not uniformly translate into increased numbers of mature, myelinating OLG during the remyelination phase. The absence of significant changes in CC1^+^, NOGO-A^+^, or Olig2^+^ oligodendroglial lineage populations following fingolimod treatment across several studies suggests that OLG survival alone is insufficient to overcome barriers to differentiation and myelin formation once demyelination is established, particularly in the context of persistent neuroinflammation within lesions (Kim et al. 2018; Hu et al. 2011; Alme et al. 2015). This dissociation between oligodendroglial lineage preservation and differentiation highlights a key limitation of S1PR modulators in promoting de novo remyelination, particularly in environments where inflammatory signalling, myelin debris, or axonal damage persists.

Nevertheless, a subset of studies reported engagement of reparative lineage dynamics, characterised by expansion of OPCs and intermediate-stage lineage populations, including BrdU^+^/Olig2^+^ proliferating oligodendroglial cells and modest increases in GST-π^+^ post-mitotic oligodendrocytes (Yazdi et al. 2015). More pronounced lineage progression was observed following treatment with JTE-013 and RP-101074, which increased both early progenitor pools (EdU^+^/PDGFRα^+^, Sox2^+^) and differentiated oligodendrocytes (EdU^+^/CC1^+^) (Seyedsadr et al. 2019; Sindi et al. 2023), suggesting that certain S1P-targeting strategies may more effectively support coordinated lineage progression. These findings align with evidence from progressive MS indicating that remyelination failure reflects not only impaired OPC recruitment, but also deficits in oligodendrocyte differentiation and survival, all governed by disease stage and lesion microenvironment (Kuhlmann et al. 2023). Together, our evaluations indicate that while S1P modulators consistently support oligodendroglial survival within myelin lesions, their role in promoting the differentiation of newly formed or existing OLG, a key remyelinating process, remains inconclusive.

### Mechanism of S1P Modulators: How Do They Work?

A key question is what mechanism underpins the effects of S1P modulators in myelin lesions. Currently available S1P modulators were originally designed as receptor-selective ligands to modulate S1PR signalling, most notably S1PR1, by inducing receptor internalisation and degradation, thereby preventing the lymphocyte egress from secondary lymphoid organs (Aoki et al. 2016; Brinkmann et al. 2010). This mechanism effectively reduces immune cell infiltration into the CNS and limits inflammatory demyelination. Preclinical studies suggest they may also affect oligodendrocyte lineage cells via S1PR5, but these mechanisms remain incompletely defined. Fingolimod shows strong evidence supporting an S1PR1-mediated immune mechanism, where conditional deletion of S1PR1 in glial fibrillary acidic protein (GFAP)-expressing astrocytes or a phosphorylation defect in the S1PR1 gene abolishes fingolimod’s efficacy, confirming its dependence on S1PR1 (Choi et al. 2011; Tsai et al. 2016). Although fingolimod has been proposed to exert direct CNS effects, including oligodendrocyte support and modulation of myelin membrane dynamics, these findings are largely restricted to in vitro or ex vivo systems (Miron et al. 2010), while in vivo remyelination outcomes remain inconsistent across models. Kim et al. demonstrated that fingolimod selectively downregulates S1PR1 but not S1PR5, providing a mechanistic explanation for its robust effect on preventing immune-mediated demyelination but comparatively weak effect on OPC-driven remyelination, which appears to depend on S1PR5 signalling (Kim et al. 2018). Therefore, the comparatively stronger remyelination outcomes reported for siponimod, ponesimod, and RP-101074 are consistent with direct engagement of oligodendrocyte lineage signalling via S1PR5, rather than indirect immunosuppressive effects from S1PR1 signalling alone.

Siponimod, a selective S1PR1/5 modulator, was developed to enhance CNS-specific actions while retaining immunomodulatory efficacy. Studies included in this review demonstrate preservation of OLGs and reduced demyelination, with some evidence of improved remyelination compared to fingolimod (Al-Otaibi et al. 2022). Siponimod-mediated OLG protection occurs without activation of classical remyelination pathways or direct suppression of glial inflammatory signalling in vitro, indicating that its effects may reflect S1PR5-dependent OLG survival rather than induction of OPC differentiation. Supporting this mechanism, Behrangi et al. showed that S1PR5 genetic deletion abolishes siponimod’s protective effects, while in vitro it does not suppress cytokine production in glia, indicating in vivo anti-inflammatory effects are indirect (Behrangi et al. 2022). Despite also targeting S1PR1 and S1PR5, the mechanistic profile of ozanimod appears to be dominated by S1PR1 engagement. Selkirk et al. found that unbound plasma and brain concentrations sufficient for ozanimod’s therapeutic efficacy in the EAE model exceeded the EC_50_ for S1PR1 (EC50 = 0.41 nM) but remain below that required for S1PR5 activation (EC50 = 11 nM, Table 1). This finding suggests that ozanimod’s effects are likely mediated predominantly through S1PR1 signalling, consistent with peripheral immunomodulation and possibly OLG survival, rather than direct S1PR5-mediated remyelination (Selkirk et al. 2021). These findings provide a mechanistic explanation for the modest and inconsistent remyelination observed with ozanimod and support the conclusion that S1PR1 activation alone is insufficient to drive effective remyelination.

Across the included studies, dose dependency emerged as an important determinant of therapeutic outcome, with higher doses generally associated with stronger protection against demyelination, whereas intermediate or lower doses more consistently supported remyelination. For example, 0.3 mg/kg fingolimod enhanced PLP^+^ myelination and oligodendrocyte differentiation more effectively than 1 mg/kg in the LPC model (Yazdi et al. 2015), and a lower dose of 2 mg/kg of siponimod was more protective than 6 mg/kg (Dietrich et al. 2022), although very low doses of 0.3 mg/kg were insufficient (Behrangi et al. 2022). Clear evidence for a bell-shaped dose–response relationship was provided by Sindi et al., who demonstrated that a 1 mg/kg dose of RP-101074 was more effective than 5 mg/kg at preventing visual function loss (Sindi et al. 2023). This pattern likely reflects dose-dependent differences in S1PR engagement and internalisation, where higher doses promote more extensive downregulation and bias signalling towards S1PR1-mediated immunomodulation, which may limit optimal engagement of S1PR5-dependent remyelination pathways. Moderate levels of receptor engagement may better support S1PR5-associated signalling within the oligodendrocyte lineage, whereas insufficient dosing might fail to activate either pathways (Behrangi et al. 2022).

Indeed, Table 1 highlights that several S1P modulators, including fingolimod, ozanimod, and ponesimod, exhibit greater potency towards S1PR1 relative to S1PR5. This raises the possibility that, at clinically relevant or higher concentrations, these modulators may preferentially exert S1PR1-mediated immunomodulatory effects before sufficient S1PR5 engagement is achieved. However, this interpretation should be made cautiously, as molecular binding evidence was not uniformly reported across all modulators. For example, data for RP-101074 were not available, hence RP-101075 was used as a proxy, which may not accurately reflect the pharmacological profile of RP-101074. Nevertheless, this interpretation is consistent with in vitro studies demonstrating dose-dependent effects between S1P-mediated OLG survival and OPC differentiation (Miron et al. 2008; Zhang et al. 2017).

Overall, pharmacological and genetic evidence support that S1P modulators such fingolimod and ozanimod primarily target S1PR1 for immune modulation, subsequent oligodendrocyte survival, whilst others such as siponimod primarily target S1PR5 for oligodendrocyte protection, although their capacity to drive remyelination remains largely unknown. Importantly, except for fingolimod and siponimod, several other modulators reported in the selected studies lack sufficient pharmacokinetic or genetic validation, highlighting a key gap in understanding their mechanism of action. This is particularly relevant for research compounds such as JTE-013, where limited pharmacokinetic data make it difficult to determine whether its mixed remyelination outcomes (i.e., increased myelin staining without corresponding ultrastructural confirmation via TEM) might reflect insufficient BBB penetration or inadequate receptor engagement within the CNS, further limiting interpretation of these findings.

### Future Research Perspectives

Despite significant effort in understanding S1P modulators using in vivo models of central demyelination, their efficacy in remyelination is lacking, highlighting a clear gap for future research. An appropriate experimental paradigm and treatment window in a combination with molecular, histological, and ultrastructural approaches, will enable more complete understanding as to whether S1P-based therapeutics promote de novo myelin formation or merely preserve existing myelin. While prophylactic modulation of S1PR often yields more pronounced protective effects on myelin compared to therapeutic treatments (Kim et al. 2018; Dietrich et al. 2022), such designs are predominantly used to investigate mechanisms of demyelination prevention rather than remyelination. Furthermore, currently no animal models faithfully represent the actual clinical course of MS, indicating the need to adopt complementary models for the evaluation of therapeutic efficacy. Future studies should therefore emphasise therapeutic treatment paradigms initiated after demyelination, to better model clinical scenarios and directly examine repair processes.

Notably, no studies identified in this review investigated modulation of S1P metabolism, such as targeting its synthesis through sphingosine kinases 1 and 2 (Sphk1/2) or its degradation through sphingosine 1-phosphate lyase (SPL). The level of bioactive S1P is tightly regulated through an equilibrium between its synthesis, mediated by SphK1/2, and its degradation by SPL, within a complex sphingolipid metabolic network (George and Xiao 2024; Xiao 2023; van Echten-Deckert 2023). As the terminal enzyme in the catabolic pathway of sphingolipids, SPL also regulates the degradation of sphingosines, sphingomyelins, and ceramides—all of which contribute to myelin synthesis and neural repair (Giussani et al. 2021). Despite their therapeutic potential, approaches that selectively modulate endogenous S1P levels remain limited due to the complexity of the sphingolipid metabolic network. Nonetheless, manipulating endogenous S1P synthesis or degradation represents a promising alternative strategy that may mitigate adverse effects associated with disease-modifying therapies such as lymphopenia, myocardial infarction, atrioventricular block, and bradycardia (Coyle et al. 2024). Unlike S1PR modulators, which induce irreversible receptor internalisation and degradation, endogenous S1P signalling involves receptor recycling, potentially allowing more physiologically regulated signalling (Xiao 2023). Exploration of these metabolic pathways may therefore offer a novel direction for promoting CNS repair. Finally, future research can prioritise in vivo mapping of receptor-specific downstream signalling pathways, particularly those linked to oligodendrocyte differentiation and myelin formation. Improved understanding of how different S1PR subtypes engage intracellular pathways in different lesion environments will be critical for developing more selective and effective strategies to enhance remyelination.

## Conclusion

This review for the first time systematically evaluated the effects of S1P-based therapeutics on in vivo remyelination in the mammalian CNS, with particular emphasis on myelin protection and oligodendrocyte lineage dynamics. Across all selected studies, S1P receptor modulators consistently limited demyelination; however, their capacity to promote remyelination and oligodendrocyte lineage progression was variable and somewhat inconclusive. Collectively, these findings support a hypothesis that therapeutics focusing on targeting individual S1PRs may possess limited capacity to enable oligodendrocyte survival and differentiation and ultimately myelin repair in myelin lesions where there is ongoing and often aggressive inflammation. While future research should continue to optimise siponimod and ponesimod for remyelination, this review argues the need to develop new S1P-based therapeutics, such as modulating S1P levels via its metabolic pathways, which could circumvent non-specific effects associated with non-selective receptor modulation (e.g. incomplete S1PRs internalisation). In addition, the heterogeneity of reported outcomes and remyelination phases further underscores the influence of experimental model and study designs that may complicate outcome interpretation. The findings of this review offer a framework to guide future model selection and experimental design aimed at determining remyelination outcomes for demyelinating diseases such as MS.

## Data Availability

Not applicable.
